# Comment on Minich et al. Is Melatonin the “Next Vitamin D”?: A Review of Emerging Science, Clinical Uses, Safety, and Dietary Supplements. *Nutrients* 2022, *14*, 3934

**DOI:** 10.3390/nu15061506

**Published:** 2023-03-21

**Authors:** Ryszard Pluta

**Affiliations:** Department of Pathophysiology, Medical University of Lublin, 20090 Lublin, Poland; pluta@imdik.pan.pl or pluta2018@wp.pl

I read an article by Minich D.M. et al. [1] with great interest that suggests melatonin as the “next vitamin D”. The authors claim that there are clear similarities between melatonin and vitamin D in their health effects, focusing on the data on melatonin’s mechanisms of action. The article does not provide a clear answer to the question posed in the title. In conclusion, the authors claim that “overall, melatonin is an intriguing compound, not unlike vitamin D, which is pleiotropic in activity and responsive to light-dark cycles” [1]. This study provides an opportunity to discuss the possible effects of natural substances, such as melatonin, in the prevention and/or treatment of neurodegenerative diseases such, as post-ischemic brain [2] and Alzheimer’s disease [3]. In this extensive and interesting analysis, I noticed that the authors presented the effect of melatonin on the modification of amyloid, tau protein, and neurotransmission in the brain in a very limited way; although the authors gently touch on this problem, the changes of which are significant in post-ischemic neurodegeneration [2] and in Alzheimer’s disease [3].

There is evidence that melatonin enhances the function of α-secretase and promotes the non-amyloidogenic pathway [4]. Melatonin inhibits the β-secretase function and the expression of the amyloid protein precursor, which affects the production of amyloid [3,4]. It also modulates the activity of the genes of the above proteins [3]. Melatonin inhibits the formation of amyloid oligomers and reduces their toxicity [5]. It interacts directly with amyloid and inhibits its aggregation [6]. Melatonin modifies the secondary structure of amyloid and promotes the conversion of the β-sheet into a random coil, which inhibits amyloid oligomerization and aggregation [3,4]. 

Research supports melatonin’s ability to prevent, reduce, or remove amyloid plaques from the brain, which is associated with improved spatial learning and memory [7]. A clinical trial has shown that the elimination of amyloid from the brain during sleep is significantly increased compared to the waking brain [8]. 

Moreover, the ability of melatonin to inhibit the hyperphosphorylation of the tau protein has been well documented in in vitro and in vivo studies [4,7]. In a transgenic model of Alzheimer’s disease, melatonin has been shown to lower levels of hyperphosphorylated tau protein, and exercise further reduces levels of amyloid oligomers [5]. In addition, a study of mice injected with β-amyloid peptide 1–42 into the brain to induce Alzheimer’s disease showed that they had decreased hyperphosphorylated tau protein following the administration of melatonin, resulting in improved neuronal viability [9]. Inhibition of the melatonin synthesizing enzyme 5-hydroxyindole-O-methyltransferase has also been shown to result in tau protein hyperphosphorylation and spatial memory impairment, which were reversible with melatonin administration [7]. 

Melatonin has been shown to influence the neurotransmission of the cholinergic and glutamatergic systems [7,10].

All these studies suggest that melatonin may be effective in preventing the pathology of amyloid and tau protein and modulating the metabolism of the amyloid protein precursor. I have doubts about the suggestion that melatonin is the “next vitamin D”. Does vitamin D have the therapeutic properties described above for melatonin? That would certainly reinforce the main idea of this article. Congratulations to the authors for having the courage to speak out on such a difficult and controversial topic.
